# Quantifying the colour loss of green field pea (*Pisum sativum L*.) due to bleaching

**DOI:** 10.1371/journal.pone.0221523

**Published:** 2019-08-23

**Authors:** Linda S. McDonald, Phillip A. Salisbury, Rebecca Ford, Joseph F. Panozzo

**Affiliations:** 1 Agriculture Victoria Research, Department of Jobs, Precincts and Regions, Horsham, Victoria, Australia; 2 Faculty of Veterinary and Agricultural Sciences, The University of Melbourne, Parkville, Victoria, Australia; 3 School of Environment and Science, Griffith University, Nathan, Queensland, Australia; National Bureau of Plant Genetic Resources, Pusa India, INDIA

## Abstract

Post-harvest change in the colour of green field pea (*Pisum sativum L*.) is undesirable as this impacts the visual quality and market value of the seed. To date, there is no standard, objective method to determine bleaching. Therefore, the aim of this study was to develop an objective method for scoring bleaching based on colour reflectance spectra, measured both by spectrophotometer and multispectral Image Analysis (IA). Green field pea seeds were sorted into samples of uniform colour and these were used to train the model. Spectra calculated from multispectral images (with colour bands at 405,470,530,590,660 and 850nm) were matched to the spectrophotometer output through multiple linear regression. All spectra were transformed to emphasize the wavelength regions most impacted during bleaching, following which two critical reflectance values were scaled to a single bleaching score. The bleaching assessment method was tested in a time-course experiment comprising seeds from five green-pea genotypes stored for six months. Each sample was divided into two so that half of the seeds were stored in the dark and the remainder were exposed to controlled light to exaggerate bleaching. Throughout this period, the samples were imaged at six-weekly intervals. Assessment of bleaching by the IA method agreed well with spectrophotometer measurements, achieving a Lin’s concordance statistic of 0.99 and 0.96 for the calibration and time-course samples respectively. The IA method proved more versatile because assessments could be made on individual seeds enabling the computation of bleaching uniformity within each sample. This method captured differences between genotypes in the extent, rate and uniformity of bleaching. All genotypes exhibited susceptibility to bleaching when stored under the controlled light conditions. Excell was observed to be the most susceptible genotype with the greatest bleaching-rate and OZB1308 displayed the most colour-stability.

## Introduction

Green pea is one of the major market classes of the *Pisum sativum L*. pulse grain family [1] and commonly used as a high-protein food source for human consumption as well as livestock feed. Canada is the largest producer and exporter of field pea globally [2] and according to the Canadian Grain Standards Guide [3], field pea is classified as either ‘green pea’ or ‘other than green pea’ and then subsequently assessed for other quality traits. This classification emphasises the relative importance of the green pea type in comparison with the other field pea market classes.

Bleaching of green field pea adversely affects the visual quality of the grain and therefore also its market value. Bleaching is a discoloration of the pea seed as chlorophyll degrades, causing seed colour to fade toward lighter green and ultimately yellowish-cream [4]. The colour of agricultural food products is often closely related to compositional quality [5–7] and colour-uniformity is considered particularly important for marketability [8–10]. The industry standard maximum allowance (percent by weight) for bleached green pea varies between countries. Australian guidelines indicate a maximum of 1% off-colour seed for a field pea sample to achieve No. 1 grade [11] and in Canada the maximum allowance is 2% bleached seed [3]. Bleaching is therefore undesirable as it can vary widely within a sample and this decreases colour uniformity and visual appeal of the seed [4]. It is generally thought that bleaching has no effect on the seed viability [12], however Atak, Kaya [13] conducted a study of three green pea genotypes and found that the darker seed had a higher vigour and germination rate than lighter seed.

Bleaching can occur pre-harvest once the seed has reached physiological maturity, particularly if the field pea plant is subjected to wet and dry soil-moisture cycles or the seed is exposed to light during the final desiccation phase [14–16]. Therefore, delayed harvesting increases the risk of bleaching [17, 18]. Bleaching can also occur post-harvest due to sub-optimal storage conditions such as extended storage periods and exposure to high temperatures, high humidity or high light intensity [4, 19, 20].

While bleaching is a considerable marketing issue, the standard assessment technique is subjective and tedious. This involves a visual estimation of the extent of discoloration across individual seed lots and at times, manual dehulling of individual seeds to inspect cotyledon colour [3]. Due to the impact that bleaching has on the market classification of field pea quality, it is the subject of ongoing plant breeding to develop cultivars which are resistant to bleaching. A machine-vision based scoring system would improve assessment efficiency and provide an objective measure of the distribution and extent of bleaching. A small number of studies which address the associated genetic inheritance and enzyme activity have quantified bleaching through various colour scales. However, there is a lack of consistency and formality among the methods. Gubbels and Ali-Khan [14] used a visual comparison of the seed to colour-charts, giving each sample a number between 1, for bleached seed, and 9, for dark green. This method is intuitive but highly subjective and prone to human-error. McCallum, Timmerman-Vaughan [15] used video image analysis in the YUV colour space, Ubayasena, Bett [20] used the Hunter Lab “a” (red-green) colour component to assess the extent of bleaching and Steet and Tong [21] used Commission Internationale de l'Eclairage (CIE) a* (red-green) to determine greenness and relate this to chlorophyll content in pureed green pea. The YUV colour space is objective but device-dependent and therefore not easily adopted as a repeatable standard method. The Hunter a and CIE a* scales are objective and device-independent; however, bleaching does not occur linearly with respect to either scale nor does a single component of each colour space fully capture the field pea colour change.

The CIE L*a*b* colour space is the most commonly used method for assessing pulse grains. It is intuitive and objective, designed to linearly align with human-perceived colour. For green pea, typically CIE L* values are between 50 and 66, CIE a* between -4 and -1 and CIE b* between 8 and 15 [22]. As green pea seeds bleach, their L* value increases, a* decreases in magnitude and b* increases; i.e. the seed becomes simultaneously lighter, less green and more yellow. The standard measure for assessing colour changes using the CIE L*a*b* space is ΔE; calculated as the Euclidean distance between two points in the colour space. However, since the changes in CIE L*, a* and b* are not linear and because different genotypes exhibit varying tones of green and yellow/cream as they bleach, an absolute ΔE value is not intuitive for scoring bleaching.

Digital image analysis has been explored for colour assessment of many agricultural food products [9, 23, 24]. The advantages of assessing colour traits by IA are many; particularly, it is rapid, objective, repeatable and non-destructive and the high spatial resolution obtained enables detailed assessments [10]. Changes in image colour have been examined to indicate ripeness of fruits such as strawberries [25] and apples [26] and colour features have been used to identify types of green vegetables before and after heat treatment [27]. Furthermore digital images of lentils were analysed by Dell'Aquila [28] to indicate seed viability based on browning of the seed-coat. Imaging analysis models based on digital colour features have also been applied to leaves and crop canopies to determine chlorophyll and biomass [28] and to indicate macronutrient deficiencies in plants [29].

The objective and consistent nature of image analysis and its usefulness in accurately and rapidly detecting and assessing colour changes in food products makes it an attractive method for assessing bleaching in field pea providing that image colour can be consistently calibrated. Therefore, the aims of this study were to: (1) Develop an objective model based on visible reflectance spectral analysis to determine bleaching scores for green field pea samples; (2) Develop an image analysis method, based on multispectral images, to replicate the spectral analysis bleaching scores and enable assessment of bleaching at a single seed level; and (3) Apply the image analysis method to quantify bleaching score, rate and uniformity of five green pea genotypes subjected to exaggerated bleaching conditions over a period of six months.

## Materials and methods

All seed samples were sourced from a field trial grown in western Victoria (Australia) during the 2015 growing season. There were 12 field plots for each of five green pea genotypes (Excell, OZB1308, OZB1310, OZB1315 and OZB1324), totalling 60 samples. This study comprised two sets of sample-data referred to herein as the time-course data and the colour-sorted data. These data sets are detailed in the sections following.

### Colour-sorted samples and data collection

Each of the 60 green pea samples was subsampled twice (approximately 150–200 seeds per subsample). One subsample was stored in the dark to avoid bleaching effects and the other was exposed to accelerated-bleaching conditions. These were stored in a single layer in a clear, 90mm diameter petri dish under fluorescent light with the mean intensity of 7500lx. The samples were stored for a period of 9 months beginning 58 days after harvest. The position of samples on the light shelf was re-randomised at fortnightly intervals. At the completion of the nine month trial, each of the 120 green pea samples (60 stored in the dark and 60 stored in the light) was sorted by visual inspection into groups of like-colours. In total there were 145 colour-sorted samples. Multispectral images, with colour bands at 405, 470, 530, 590, 660 and 850nm, were collected for all samples using an EyeFoss^™^ (Foss Analytical, Hoganas, Sweden). Each sample was also measured for colour reflectance spectra by spectrophotometer (Minolta CM-5, Konica Minolta). Spectrophotometric measurements were taken through a glass petri dish (aperture 30mm, D65 illuminant, reflectance, spectral component excluded). Spectral data was collected as the mean of three sets of triplicate measurements and stored as discrete reflectance values recorded every 10nm from 360nm to 740nm. The data collected on the colour-sorted samples was used to develop and calibrate a model for objectively scoring bleaching based on colour reflectance spectra and to develop an image analysis method for scoring bleaching. Development of these methods is outlined in the following four sections.

### Time-course samples and data collection

Seeds were collected from three plots of each of the five green pea genotypes in the source field trial. The 15 samples were then subsampled twice (approximately 150–200 seeds per subsample) and stored under the same light and dark conditions described for the colour-sorted samples. The time-course samples were stored for a period of six months and imaged every six weeks using an EyeFoss^™^. At these same time intervals, colour reflectance spectra were also measured for each sample by spectrophotometer with the same instrument settings as used for the colour-sorted samples.

### Development of the bleaching-score model

The bleaching-score model was developed with the aim of producing a scale, ranging from 0 to 100, to quantify the extent of bleaching where 0 represented dark green seed and 100 represented completely bleached seed. Development of this model was based on spectrophotometric data of the colour-sorted green pea samples.

**Fig 1:**
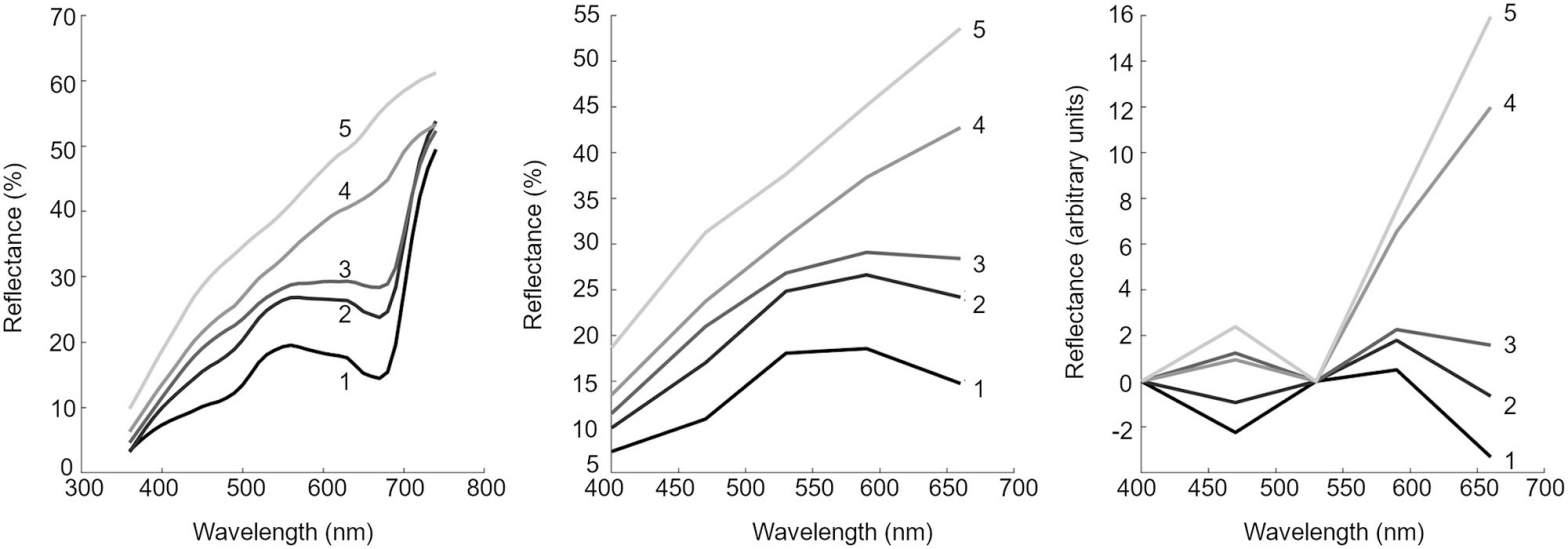
Spectral response of bleaching. Example spectra of five green pea genotypes at various stages of bleaching. Dark line (1) represented the least bleached and lightest grey line (5) represented the most bleached. (a) Original spectra measured by spectrophotometer; (b) Spectra in (a) sampled at 400,470,530,590 and 660nm; (c) Spectra at 400,470,530,590 and 660nm transformed according to the method outlined in the previous section.

Discrete colour reflectance spectra were reduced to five reflectance values, measured at 400, 470, 530, 590 and 660nm (Fig 1b), corresponding to the colour bands of the multispectral images. On inspection of the spectra it was noted that there were two regions, centred around 470nm and 590nm, where the spectra distinctly changed shape in response to bleaching colour changes. The reduced spectra were subsequently transformed to enhance the detection of this response. This transformation comprised a linear slope adjustment, to create a zero-overall gradient between 440 and 530nm, and a shift such that the transformed reflectance values at 530nm were all zero (Fig 1c). Two critical values, *C*1 and *C*2, were then calculated as a linear scaling of the transformed spectra at 470nm and 590nm, respectively (Eqs 1 and 2). The bleaching score, *B*, was then calculated as the average of the two critical values and scaled such that *B* was in the range [0,100]; *B* = 50(*C*1 + *C*2).

$$C1=\alpha_{1}T(470nm)+\beta_{1}$$ (1)

$$C2=\alpha_{2}T(590nm)+\beta_{2}$$ (2)

Where *T*(*λ*)is a transformed spectrum, in the form of the spectra depicted in Fig 1c, and *α*_1_, *α*_2_, *β*_1_ and *β*_2_ are constants to be determined such that *C*1 and *C*2 are in the range [0,1].

### Image processing to obtain single seed colour spectra

The EyeFoss^™^ is an instrument which captures multispectral images of individual seeds as they travel along a conveyor [30]. Therefore, for each green pea sample analysed through the EyeFoss there were as many multispectral images generated as there were seeds. Images were processed through Matlab and Image Processing Toolbox R2018b (The Mathworks Inc., Natick, Massachusetts, United States) software. Multispectral images were segmented to identify the seed region in each image and this region was then further eroded by five pixels around the boundary to circumvent interference of boundary-shadowing on the seed colour. For each seed, a discrete colour spectrum was computed; this comprised the 25^th^ percentile pixel intensity value of each of the six image colour bands within the seed region of the image. The 25^th^ percentile value was used instead of the mean or median, to ensure that the representative colour of any given seed was not impacted by seed regions, such as the hilum or crease, which are naturally lighter in colour independent of bleaching. Discrete spectral values were then transformed through Multiple Linear Regression (MLR) models to match image intensity values to the reflectance units of the spectrophotometer output. The MLR models were constructed using the spectrophotometer output and the mean image-derived spectral values of the colour-sorted green pea samples. Dependent and independent variables of these MLR models are listed in Table 1. Through this transformation, only the first five transformed colour bands were retained as a part of the image-derived spectra, since the 6^th^ colour band (850nm) was outside the range of the spectrophotometer measurements.

**Table 1:** Modelling the spectrophotometer output.

Wavelength of modelled reflectance value	Independent Variables *	Dependent Variable **
400nm	405nm and 470nm	400nm
470nm	405nm, 470nm and 530nm	470nm
530nm	470nm, 530nm and 590nm	530nm
590nm	530nm, 590nm and 660nm	590nm
660nm	590nm, 660nm and 850nm	660nm

MLR input model variables for transforming pixel intensity values to the reflectance units of the spectrophotometer.

* Independent variables are the pixel intensity values of listed-wavelength image colour-band

** Dependent variables are the spectrophotometer-measured reflectance values at the listed wavelengths

### Quantifying bleaching by image analysis

Discrete image spectra, calculated for each seed, were transformed in the same manner as the spectrophotometer spectra described earlier. Critical values *C*1 and *C*2 were then calculated according to Eqs 1 and 2 and the bleaching score for each individual seed was subsequently calculated. The bleaching score for each sample was taken as the average of all the individual-seed bleaching scores within that sample.

### Quantifying bleaching in the time course samples

Time course bleaching scores were predicted by image analysis for all the seeds in each sample. The rate of bleaching was measured by regression analysis for each genotype as the gradient of the mean sample bleaching scores across all measurement dates. Uniformity, *U*_*B*_, of bleaching was calculated for each genotype as $U_{B}=1-\frac{{IPR}_{B}}{100}$, where *IPR*_*B*_ is the inter-percentile range (calculated as the 90^th^ percentile minus the 10^th^ percentile) of bleaching scores. All statistical analyses were computed through the use of GenStat (18^th^ Edition) software.

## Results and discussions

### Bleaching score model and image processing

#### Image processing results

Digital-image spectral-reflectance values calculated through MLR models highly correlated with the spectrophotometer output for both the colour-sorted sample set (which was used to calibrate the MLR models) and the time-course sample set (Table 2). The time-course samples were less uniform in colour than the colour-sorted samples and this had a small impact on the accuracy of reflectance predictions, resulting in a slightly lowered Lin’s concordance statistic for each of the colour bands.

**Table 2:** Performance of pixel intensity transformation models.

Colour-band wavelength	Sample Set	R^2^	Lin’s concordance statistic	Lin’s concordance 95% C.I.
400nm	Colour-sorted	0.99	0.99	(0.98, 0.99)
470nm	Colour-sorted	0.99	0.99	(0.99,0.99)
530nm	Colour-sorted	0.99	0.99	(0.98, 0.99)
590nm	Colour-sorted	0.99	0.99	(0.99,0.99)
660nm	Colour-sorted	0.99	0.99	(0.99, 1.00)
400nm	Time-course	0.99	0.99	(0.98, 0.99)
470nm	Time-course	0.98	0.98	(0.98, 0.99)
530nm	Time-course	0.98	0.97	(0.96, 0.98)
590nm	Time-course	0.98	0.97	(0.96, 0.98)
660nm	Time-course	0.98	0.96	(0.95, 0.97)

Correlation coefficients and Lin’s concordance test statistics for the MLR models developed to transform pixel intensity values to reflect reflectance units of the spectrophotometer.

#### Selection of critical value calculation constants

**Fig 2:**
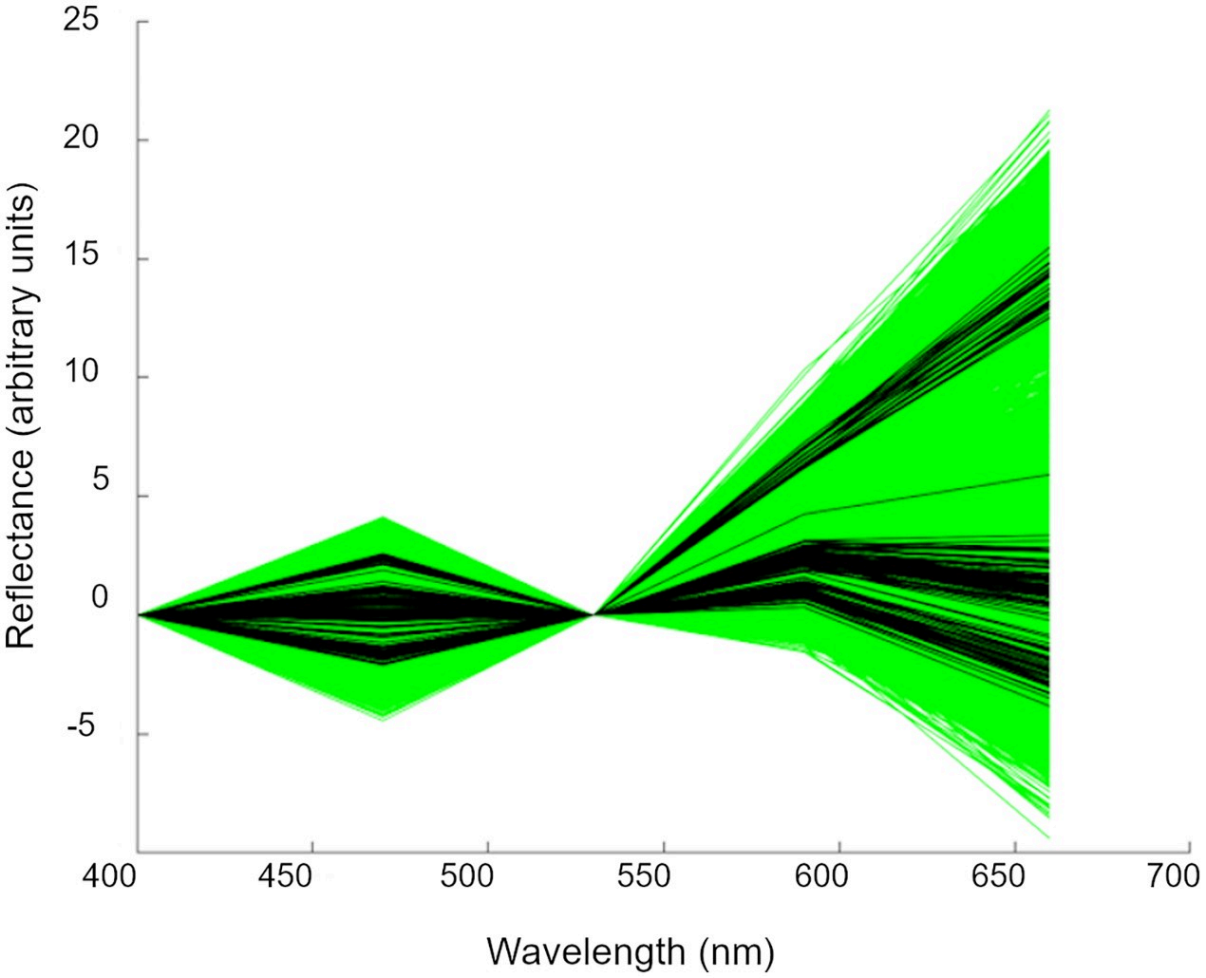
Transformed spectral range. Transformed image spectra of single seeds (green lines, n = 11309) and whole samples (black lines, n = 145). The single seed values are more extreme than the whole sample values.

In order to calculate the bleaching scores for the colour-sorted and time-course samples, the critical value coefficients, (*α*_1_, *α*_2_, *β*_1_ and *β*_2_ from Eqs 1 and 2) were determined using the maximum and minimum values of the single-seed, image-derived, spectral reflectance values (Fig 2) at wavelengths of 470nm and 590nm, since these were more extreme than the whole-sample spectral values (which were an average of the single-seed values). The coefficients were calculated according to Eqs 3, 4, 5 and 6 (Table 3).

**Table 3:** Critical value computation.

Constant	Value
α_1_	0.1204
α_2_	0.0858
β_1_	-0.5076
β_1_	-0.1342

Constant coefficient values for calculating critical values (Eqs 1 and 2)

$$\alpha 1=\frac{1}{Max470-Min470}$$ (3)

$$\alpha 2=\frac{1}{Max590-Min590}$$ (4)

$$\beta 1=\frac{-Min470}{Max470-Min470}$$ (5)

$$\beta 2=\frac{-Min590}{Max590-Min590}$$ (6)

*Max470* and *Min470* are the maximum and minimum transformed spectral reflectance values of single seed observed at 470nm. Similarly, *Max590* and *Min590* are the maximum and minimum values at 590nm (Fig 2).

To apply this bleaching score model on images taken from another instrument, the coefficients in Table 3 would remain with the same values, however the MLR model transformations of pixel intensity values would have to be calculated specifically for each imaging instrument.

#### Results of the bleaching model

**Fig 3:**
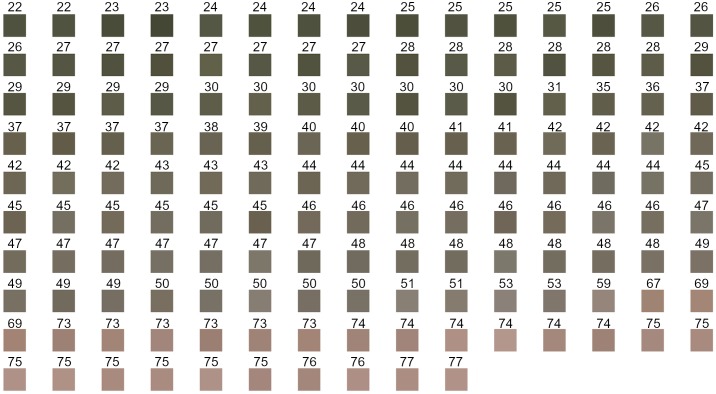
Bleaching score chart. Illustration of bleaching scores for the colour-sorted samples. The number above each square was the bleaching score assigned for that square.

**Fig 4:**
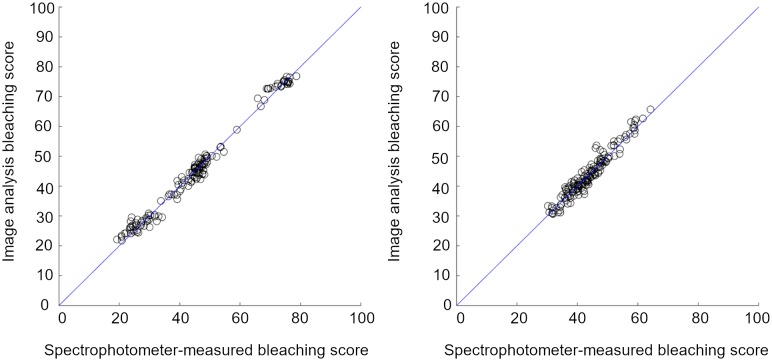
Performance of image-based bleaching scores. Bleaching scores assessed by image analysis highly correlated with bleaching scores measured by colour spectrophotometer for (a) the colour sorted samples, R^2^ = 0.99, and (b) the time-course samples, R^2^ = 0.96. Blue lines represent the one-to-one relation.

The bleaching scores depicted in Fig 3, are linearly related to the transformed spectral values at 470nm and 590nm. Reflectance values at these wavelengths highly correlated between the spectrophotometer output and image predictions (Table 2). Therefore, it was expected that bleaching scores predicted through the image analysis method would be closely related to the bleaching scores calculated from the spectrophotometer output (Fig 4 and Table 4). Image analysis predictions of bleaching scores for the time-course samples had a greater RMS error than those for the colour-sorted samples (Table 4). This is likely due to the greater range of colours within each time-course sample and therefore more variability in the colour of the seed faces which were assessed by the spectrophotometer and those which were imaged.

**Table 4:** Performance of bleaching score prediction models based on image analysis.

Sample set	Lin’s Concordance statistic	Lin’s Concordance 95% C.I.	RMSE	R^2^
Colour-sorted	0.99	(0.99, 1.00)	1.90	0.99
Time-course	0.96	(0.95, 0.97)	2.00	0.97

Correlation coefficients, Lin’s concordance test statistics and Root Mean Square Error (RMSE)values for image predictions of bleaching scores compared with spectrophotometer-derived bleaching scores.

### Determining time-course bleaching response

The time-course study was designed to assess bleaching scores at regular intervals throughout the storage period in order to quantify rates of bleaching and changes in bleaching uniformity within each genotype. Exposure of green pea to light is known to increase the rate of bleaching [4] so storing samples under light would be expected to accelerate bleaching as compared to storing samples in the dark. Accordingly, genotypes with greater resistance to bleaching would be identified. The results of this experiment are outlined in Tables 5 and 6 for bleaching scores and bleaching uniformity assessments respectively.

**Table 5:** Rate of bleaching.

Genotype	Storage	Mean bleaching score at T1	Rate of bleaching*	Rate of bleaching 95% C.I.	p-value
OZB1315	Dark	31.57± 0.67	0.63	(0.11, 1.15)	0.02
Excell	Dark	41.94 ± 0.47	0.32	(-0.10, 0.74)	0.13
OZB1310	Dark	38.84 ± 0.92	0.69	(0.12, 1.25)	0.02
OZB1308	Dark	37.75 ± 1.14	0.60	(0.12, 1.07)	0.02
OZB1324	Dark	45.52 ± 0.75	0.09	(-0.50, 0.68)	0.74
OZB1315	Light	32.24 ± 1.01	1.85	(1.17,2.54)	<0.001
Excell	Light	41.38 ± 0.54	5.49	(4.71, 6.256)	<0.001
OZB1310	Light	39.62 ± 0.84	3.46	(2.91, 4.00)	<0.001
OZB1308	Light	38.34 ± 0.81	2.82	(2.18, 3.48)	<0.001
OZB1324	Light	45.00 ± 0.83	3.65	(2.80, 4.50)	<0.001

Regression analysis on mean of single-seed bleaching scores

* Rate of bleaching was calculated as the average change in bleaching score over a 6-week period

**Table 6:** Uniformity of bleaching.

Genotype	Storage	Mean uniformity score at T1 **	Uniformity gradient* (x10^-3^)	Uniformity gradient (x10^-3^) 95% C.I.	p-value
OZB1315	Dark	0.69 ± 0.01	-2.06	(-7.24, 3.12)	0.41
Excell	Dark	0.77±0.03	0.62	(-17.87, 9.10)	0.94
OZB1310	Dark	0.66 ± 0.01	0.86	(-4.06, 5.77)	0.71
OZB1308	Dark	0.71 ± 0.01	4.04	(-4.65, 12.73)	0.33
OZB1324	Dark	0.74 ± 0.01	-2.62	(-1.99, 4.60)	0.75
OZB1315	Light	0.67 ± 0.01	-42.79	(-53.31, -32.26)	< .001
Excell	Light	0.74 ± 0.02	-30.74	(-47.23, -14.25)	0.001
OZB1310	Light	0.66 ± 0.01	-47.04	(-54.16, -39.91)	< .001
OZB1308	Light	0.71 ± 0.01	-32.96	(-40.79, -25.12)	< .001
OZB1324	Light	0.76 ± 0.02	-35.54	(-49.70, -21.32)	< .001

Regression analysis on bleaching uniformity scores

* Uniformity gradient was calculated as the average change in mean uniformity over a 6-week period

** Averaged across the three field-plot samples of the same genotype

The rate of bleaching for three of the genotypes (OZB1308, OZB1315 and OZB1310) indicated significant (p<0.05) bleaching of the seed over the storage period, i.e., the bleaching gradient was greater than zero (Table 5). However, since the magnitude of the gradients were small, this would likely be invisible to the human eye. By contrast, all samples stored under light showed a significantly high and visible shift toward bleaching over time. Therefore, each genotype exhibited some susceptibility to bleaching under these storage conditions. Excell had the steepest bleaching gradient and was the most heavily weighted toward the completely-bleached end of the scale at the completion of the storage period. Excell also had the least negative gradient for uniformity, suggesting that the colour, during bleaching, remained more uniform than for the other genotypes assessed. OZB1315 showed some resistance to bleaching overall (i.e. mean bleaching score did not shift far over the time-course study), however the uniformity of bleaching decreased at a greater rate than for all other genotypes, except OZB1310, demonstrating a tendency of OZB1315 to bleach unevenly. OZB1310 also bleached non-uniformly, maintaining a small proportion of dark green seed throughout the storage period. OZB1308 displayed the most colour stability. The rate of bleaching for OZB1308 was less than for OZB1324, OZB1310 and Excell and it generally maintained a far more uniform colour distribution than the other genotypes, making it the most bleaching-resistant of the five genotypes.

## Conclusion

Currently, there is no standard objective measure of seed-bleaching and the grain industry often relies on visual classifications which are highly subjective. Bleaching of colour in green field pea impacts the perceived quality, therefore the ability to objectively measure the extent and uniformity of bleaching through image analysis provides a quantitative tool to investigate this phenomenon. This study presented objective methods for assessing bleaching based on visible reflectance spectra (spectrophotometer measurements) and digital image analysis. These methods were successfully applied to quantify the extent of bleaching in five green pea genotypes. The image analysis method was developed to score individual seeds which subsequently enabled the quantification of bleaching-uniformity throughout each sample. The methods presented in this study provide a mechanism to examine links between bleaching and other seed qualities (e.g. seed hardness and seed composition) and to study the impact of storage conditions and agronomic practices, such as timing of harvest, on bleaching responses. The application of a high-throughput, image-based method will allow for the quantification of genetic and environmental effects on colour and bleaching. This will lead to the development of germplasm with optimal market quality.
